# *ABCA12* Frameshift Deletion in Domestic Cats With Ichthyosis Fetalis

**DOI:** 10.1111/vde.70043

**Published:** 2025-12-15

**Authors:** Jeanna M. Blake, Melissa P. Swan, Kari J. Ekenstedt

**Affiliations:** ^1^ Department of Basic Medical Sciences, College of Veterinary Medicine Purdue University West Lafayette Indiana USA; ^2^ Veterinary Diagnostic Laboratory, Department of Veterinary Sciences, College of Agriculture, Food and Environment University of Kentucky Lexington Kentucky USA

**Keywords:** autosomal recessive congenital ichthyoses, diffuse epidermal hyperkeratosis, *Felis catus*, harlequin ichthyosis, skin barrier, whole‐genome sequencing

## Abstract

**Background:**

Ichthyosis fetalis (IF), also known as harlequin ichthyosis, is a rare and often fatal autosomal recessive congenital skin disorder. It is characterized by thickened, hard skin plaques and deep skin fissures that limit mobility and cause malformations of the eyes, lips and ears. Affected individuals are highly susceptible to life‐threatening infections due to the disruption of the skin's protective barrier. To date, IF and its genetic basis have not been described in domestic cats.

**Objectives:**

To characterize the gross clinical, histopathological and genetic features of IF in two stray, random‐bred domestic short‐hair (DSH) littermates.

**Animals:**

Two deceased female neonatal DSH kittens, both exhibiting sparse hair and deep fissures exposing the underlying dermis. One unrelated neonatal kitten with normal skin and hair was included as a control for comparison, along with 140 feline population samples from unrelated domestic cats of various breeds.

**Materials and Methods:**

Gross clinical examination, histopathological analysis, whole‐genome sequencing and population genotyping were performed.

**Results:**

Gross clinical and histopathological evaluations confirmed a diagnosis of IF in both affected kittens. Genetic analysis identified a homozygous one base pair deletion in *ABCA12*, resulting in a frameshift and predicted loss of function of the encoded protein. Genotyping of 140 unrelated cats revealed that all were homozygous for the wild‐type allele.

**Conclusions and Clinical Relevance:**

Variants in *ABCA12* have been implicated previously in IF in humans, cattle and mice. This study provides the first description of IF in domestic cats and identifies a pathogenic *ABCA12* frameshift variant as the likely genetic cause.

## Abstract 2

**Background**—Ichthyosis fetalis (IF), also known as harlequin ichthyosis, is a rare and often fatal autosomal recessive congenital skin disorder. It is characterized by thickened, hard skin plaques and deep skin fissures that limit mobility and cause malformations of the eyes, lips and ears. Affected individuals are highly susceptible to life‐threatening infections due to the disruption of the skin's protective barrier. To date, IF and its genetic basis have not been described in domestic cats. **Objectives**—To characterize the gross clinical, histopathological and genetic features of IF in two stray, random‐bred domestic short‐hair littermates. **Conclusions and Clinical Relevance**—Variants in *ABCA12* have been implicated previously in IF in humans, cattle and mice. This study provides the first description of IF in domestic cats and identifies a pathogenic *ABCA12* frameshift variant as the likely genetic cause.

## ZUSAMMENFASSUNG

**Hintergrund:**

Ichthyosis fetalis (IF), auch bekannt als Harlekin‐Ichthyose, ist eine seltene und oft tödliche autosomal‐rezessive angeborene Hauterkrankung. Sie ist gekennzeichnet durch verdickte, harte Hautplatten und tiefe Hautfurchen; diese schränken die Beweglichkeit ein und verursachen Fehlbildungen der Augen, Lippen und Ohren. Betroffene Individuen sind aufgrund der Störung der schützenden Hautbarriere stark anfällig für lebensbedrohliche Infektionen. Bislang wurden IF und ihre genetischen Grundlagen bei Hauskatzen nicht beschrieben.

**Ziele:**

Ziel war es, die groben klinischen, histopathologischen und genetischen Merkmale von IF bei zwei streunenden, zufällig gezüchteten Hauskatzen‐Geschwistern mit kurzem Fell (DSH) zu charakterisieren.

**Tiere:**

Untersucht wurden zwei verstorbene weibliche neugeborene DSH‐Kätzchen, die beide spärliches Fell und tiefe Furchen zeigten, welche die darunterliegende Dermis freilegten. Zum Vergleich wurde ein nicht verwandtes neugeborenes Kätzchen mit normaler Haut und normalem Fell sowie 140 Populationsproben von nicht verwandten Hauskatzen verschiedener Rassen als Kontrollgruppe herangezogen.

**Material und Methoden:**

Es wurden eine grobe klinische Untersuchung, histopathologische Analysen, eine Ganzgenom‐Sequenzierung und eine Genotypisierung der Population durchgeführt.

**Ergebnisse:**

Die grobe klinische und histopathologische Bewertung bestätigten die Diagnose IF bei beiden betroffenen Kätzchen. Die genetische Analyse identifizierte eine homozygote Ein‐Basenpaar‐Deletion im Gen ABCA12, die zu einer Leserahmenverschiebungs‐Mutation und einem vorhergesagten Funktionsverlust des kodierten Proteins führte. Bei der Genotypisierung von 140 nicht verwandten Katzen waren alle homozygot für das Wildtyp‐Allel.

**Schlussfolgerungen und klinische Bedeutung:**

Varianten im Gen ABCA12 wurden zuvor mit IF bei Menschen, Rindern und Mäusen in Verbindung gebracht. Diese Studie liefert die erste Beschreibung von IF bei Hauskatzen und identifiziert eine pathogene Leserahmenverschiebungs‐Variante im Gen ABCA12 als wahrscheinliche genetische Ursache.

## 摘要

**背景:**

胎儿鱼鳞病(Ichthyosis fetalis, IF)，又称哈利奎鱼鳞病，是一种罕见且常致命的常染色体隐性先天性皮肤疾病。其特征包括皮肤增厚、坚硬的皮肤斑块，以及深层皮肤开裂；这些改变会限制活动，并导致眼、唇和耳部畸形。由于皮肤屏障严重受损，患病个体极易发生危及生命的感染。目前为止，尚未有关于家猫发生 IF 及其遗传基础的描述。

**研究目的:**

描述两只流浪、随机交配的短毛家猫(DSH)同窝幼猫的 IF 的肉眼临床特征、组织病理特征及遗传学特征。

**动物:**

两只已死亡的雌性新生 DSH 小猫，均表现为毛发稀疏、深层皮肤裂隙以致真皮外露。另选取一只皮肤和毛发正常的无关新生小猫作对照，同时纳入来自不同品种、共 140 只无关家猫的样本用于群体基因分型。

**材料与方法:**

进行肉眼临床检查、组织病理分析、全基因组测序及群体基因分型。

**结果:**

肉眼与组织病理学评估均证实两只患病小猫的诊断为 IF。遗传学分析发现 ABCA12 基因存在一个纯合的 1 bp 缺失突变，导致移码并使编码蛋白发生预期的功能缺失。对 140 只无关家猫的基因分型显示，它们均为野生型等位基因的纯合状态。

**结论与临床相关性:**

ABCA12 基因变异此前已被证明与人类、牛和小鼠的 IF 有关。本研究首次描述了家猫的 IF，并确定一种致病性的 ABCA12 移码突变可能是其遗传病因。

## Résumé

**Contexte:**

L'ichtyose fœtale (IF), également connue sous le nom d'ichtyose arlequin, est une maladie cutanée congénitale autosomique récessive rare et souvent mortelle. Elle se caractérise par des plaques cutanées épaisses et dures et des fissures cutanées profondes qui limitent la mobilité et provoquent des malformations des yeux, des lèvres et des oreilles. Les personnes atteintes sont très sensibles aux infections potentiellement mortelles en raison de la perturbation de la barrière protectrice de la peau. À ce jour, l'IF et sa base génétique n'ont pas été décrites chez les chats domestiques.

**Objectifs:**

Caractériser les principales caractéristiques cliniques, histopathologiques et génétiques de l'IF chez deux chatons errants de race domestique à poil court (DSH) issus de la même portée.

**Animaux:**

Deux chatons DSH nouveau‐nés décédés, tous deux présentant une pilosité clairsemée et des fissures profondes exposant le derme sous‐jacent. Un chaton nouveau‐né non apparenté, présentant une peau et une pilosité normales, a été inclus comme témoin à des fins de comparaison, ainsi que 140 échantillons de population féline provenant de chats domestiques non apparentés de différentes races.

**Matériels et méthodes:**

Un examen clinique macroscopique, une analyse histopathologique, un séquençage du génome entier et un génotypage de la population ont été réalisés.

**Résultats:**

Les évaluations cliniques et histopathologiques macroscopiques ont confirmé le diagnostic d'IF chez les deux chatons atteints. L‘analyse génétique a identifié une délétion homozygote d'une paire de bases dans le gène ABCA12, entraînant un décalage du cadre de lecture et une perte de fonction prévue de la protéine codée. Le génotypage de 140 chats non apparentés a révélé que tous étaient homozygotes pour l'allèle de type sauvage.

**Conclusions et pertinence clinique:**

Des variants du gène ABCA12 ont déjà été impliqués dans l'IF chez l'homme, le bétail et la souris. Cette étude fournit la première description de l'IF chez les chats domestiques et identifie un variant pathogène du gène ABCA12 comme cause génétique probable.

## 要約

**背景:**

胎児性魚鱗癬(IF)は、道化師様魚鱗癬としても知られ、稀でしばしば致死的な常染色体劣性遺伝性の先天性皮膚疾患である。肥厚した硬い局面および深い皮膚亀裂を特徴とし、運動能力を制限し、眼、唇、耳の奇形を引き起こす。罹患個体は、皮膚の保護バリアが破壊されるため、生命を脅かす感染症に非常に罹患しやすくなる。現在まで、家猫におけるIFとその遺伝学的根拠は報告されていません。

**目的:**

本研究の目的は、野良猫でランダムブリードのドメスティック・ショートヘア(DSH)の同腹仔2頭におけるIFの肉眼的臨床的、組織病理学的、および遺伝学的特徴を明らかにすることであった。

**対象:**

死亡した雌のDSH新生子猫2頭。どちらも毛がまばらで、真皮が露出する深い亀裂が見られた。血縁関係のない新生子猫1頭(正常な皮膚と被毛を有する)を比較対照として用い、血縁関係のない様々な品種の飼い猫140頭の集団サンプルも含めた。

**材料と方法:**

肉眼的臨床検査、組織病理学的解析、全ゲノムシークエンシング、集団遺伝子型解析を実施した。

**結果:**

肉眼的臨床初見および組織病理学的評価により、罹患した2頭の子猫はいずれもIFと診断した。遺伝子解析により、ABCA12遺伝子にホモ接合性の1塩基対欠失が同定され、フレームシフトおよびコードされているタンパク質の機能喪失が予測された。血縁関係のない140頭の猫の遺伝子解析では、全ての猫が野生型対立遺伝子のホモ接合体であることが明らかになった。

**結論と臨床的意義:**

ABCA12遺伝子の変異は、これまでヒト、ウシ、マウスのIFに関連していることが示唆されてきた。本研究は、家猫におけるIFに関する初めての報告であり、病原性のABCA12フレームシフト変異が遺伝学的原因である可能性が高いことを明らかにした。

## Resumo

**Contexto:**

A ictiose fetal (IF), também conhecida como ictiose arlequim, é uma doença congênita da pele, autossômica recessiva, rara e frequentemente fatal. É caracterizada por placas cutâneas espessas e endurecidas e fissuras profundas na pele, estas limitam a mobilidade e causam malformações nos olhos, lábios e orelhas. Os indivíduos afetados são altamente suscetíveis a infecções com risco de vida devido à ruptura da barreira protetora da pele. Até o momento, a IF e sua base genética não foram descritas em gatos domésticos.

**Objetivos:**

Investigar as características clínicas, histopatológicas e genéticas da IF em dois gatos domésticos de pelo curto (DSH) sem raça definida, irmãos de ninhada.

**Animais:**

Duas gatinhas DSH neonatas falecidas, ambas apresentando pelos esparsos e fissuras profundas expondo a derme subjacente. Um gatinho neonato não aparentado, com pele e pelos normais, foi incluído como controle para comparação, juntamente com 140 amostras populacionais felinas de gatos domésticos não aparentados de várias raças.

**Materiais e Métodos:**

Foram realizados exame clínico macroscópico, análise histopatológica, sequenciamento de genoma completo e genotipagem populacional.

**Resultados:**

As avaliações clínicas macroscópicas e histopatológicas confirmaram o diagnóstico de IF em ambos os gatinhos afetados. A análise genética identificou uma deleção homozigótica de um par de bases no gene ABCA12, resultando em uma mudança na fase de leitura e na perda de função prevista da proteína codificada. A genotipagem de 140 gatos não relacionados revelou que todos eram homozigóticos para o alelo selvagem.

**Conclusões e Relevância Clínica:**

Variantes no gene ABCA12 já foram implicadas em IF em humanos, bovinos e camundongos. Este estudo fornece a primeira descrição de IF em gatos domésticos e identifica uma variante patogênica de mudança na fase de leitura do gene ABCA12 como a provável causa genética.

## RESUMEN

**Introducción:**

La ictiosis fetal (IF), también conocida como ictiosis arlequín, es un trastorno cutáneo congénito autosómico recesivo, poco frecuente y a menudo mortal. Se caracteriza por placas cutáneas engrosadas y duras, y profundas fisuras cutáneas que limitan la movilidad y causan malformaciones en ojos, labios y orejas. Los individuos afectados son muy susceptibles a infecciones potencialmente mortales debido a la alteración de la barrera protectora de la piel. Hasta la fecha, la IF y su base genética no se han descrito en gatos domésticos.

**Objetivos:**

Caracterizar las características clínicas, histopatológicas y genéticas de la IF en dos gatos domésticos callejeros de pelo corto (DSH) de progenitores al azar de la misma camada.

**Animales:**

Dos gatitas DSH neonatales fallecidas, ambas con pelo ralo y profundas fisuras que exponen la dermis subyacente. Se incluyó como control una gatita neonatal no emparentada con piel y pelo normales, junto con 140 muestras de la población felina de gatos domésticos no emparentados de diversas razas.

**Materiales y métodos:**

Se realizó un examen clínico macroscópico, análisis histopatológico, secuenciación del genoma completo y genotipificación poblacional.

**Resultados:**

Las evaluaciones clínicas e histopatológicas macroscópicas confirmaron el diagnóstico de IF en ambos gatitos afectados. El análisis genético identificó una deleción homocigótica de un par de bases en ABCA12, lo que resultó en un desplazamiento del marco de lectura y la pérdida de función prevista de la proteína codificada. La genotipificación de 140 gatos no emparentados reveló que todos eran homocigotos para el alelo silvestre.

**Conclusiones y relevancia clínica:**

Las variantes en ABCA12 se han implicado previamente en la IF en humanos, ganado vacuno y ratones. Este estudio proporciona la primera descripción de la IF en gatos domésticos e identifica una variante patógena con desplazamiento del marco de lectura de ABCA12 como la probable causa genética.

## 1 Introduction

Autosomal recessive congenital ichthyoses (ARCI) comprise a large and heterogenous group of inherited skin disorders characterized by widespread dryness, thickening, and scaling of the skin. Variants in numerous genes have been associated with ARCI in both humans and other mammals [1, 2, 3, 4, 5, 6, 7, 8, 9, 10, 11]. Three major clinical phenotypes of ARCI have been reported: congenital ichthyosiform erythroderma (CIE), lamellar ichthyosis (LI) and harlequin ichthyosis (HI) [11]. CIE is characterized by fine white scales and widespread erythroderma [11, 12], while LI presents with large, dark, pigmented scales in the absence of erythroderma [11]. HI, also referred to as ichthyosis fetalis (IF), is the most severe form of ARCI. It is characterized by profound skin abnormalities including malformations of the eyes (ectropion) and lips (eclabium), rudimentary ears, alopecia, deep reddened skin fissures, and thick armour‐like, diamond‐shaped skin plaques covering the entire body, resulting in severe hyperkeratosis [13, 14, 15, 16]. The IF phenotype is highly specific for loss‐of‐function genetic defects (e.g., non‐sense, frameshift, splice‐site and truncating variants) in the *ATP‐binding cassette subfamily A member 12* (*ABCA12*) gene, which encodes a lipid transporter essential for normal skin barrier function [13, 17]. In contrast, missense variants in *ABCA12* are generally associated with milder ARCI phenotypes, including CIE or LI [18, 19, 20]. Additionally, pathogenic variants in several other genes besides *ABCA12* have been implicated in cases of CIE and LI [2, 3, 4, 5, 21, 22].

In humans, IF is an extremely rare, phenotypically severe, and historically lethal inherited nonsyndromic skin disorder. Affected neonates often are stillborn or die shortly after birth due to loss of‐skin barrier function, which often leads to complications such as excessive trans epidermal water loss, electrolyte imbalance, impaired thermal regulation, and a significantly increased risk of developing infection and sepsis [23, 24]. The genetic etiology of IF is well‐characterized, with pathogenic *ABCA12* variants described in humans [16, 17, 20, 24] (OMIM 242500), as well as in cattle [1, 25, 26] (OMIA 002238‐9913), pig models [27] (OMIA 002238‐9823) and murine models [28]. To date, over 50 IF‐causing *ABCA12* variants have been identified in humans, most of which are predicted to result in premature protein truncation, highlighting the allelic heterogeneity of this condition [13, 17, 20, 29, 30]. ABCA12 is a keratinocyte transmembrane lipid transporter necessary for the movement of lipids across cell membranes via lamellar granules into the stratum corneum (SC) of the epidermis, where they form extracellular lipid layers that are essential for maintaining the skin's barrier function [29].

Ichthyosis fetalis has not been reported in companion animals and has never been documented in cats. The purpose of this study was to provide a detailed gross clinical phenotypic, pathological, and genetic characterization of two random‐bred kitten littermates diagnosed with IF, and to describe the results of a population screening for the *ABCA12* frameshift variant identified in these cases.

## 2 Materials and Methods

### 2.1 Ethics

All procedures were followed in accordance with Purdue University's Institutional Animal Care and Use Committee (IACUC no. 1901001840). The two deceased stray cats and the control included in this study were not euthanized for the purpose of this research; they were used post‐mortem in compliance with IACUC approved protocols. All additional cats in this study were privately owned pet cats.

### 2.2 Animals

Two stray, female, neonatal, random‐bred domestic short‐hair (DSH) kittens presenting with skin lesions resembling thermal burns were brought to the City of Chicago Animal Care and Control as a suspected neglect or abuse case. One kitten was found deceased, and the other kitten was humanely euthanized as a result of the severity of its condition. Both kittens were transferred to the Animal Disease Diagnostic Laboratory at Purdue University for full necropsy and diagnostic evaluation.

For histopathological comparison, abdominal skin from a single, unrelated, 5‐day‐old neonatal kitten, clinically unaffected by IF was used as a control. This kitten, which had been orphaned and was being hand‐raised out of a local shelter, died from unknown natural causes while being fostered. IACUC approval was not required for the use of any of the three kittens in this study, as all were deceased before research involvement.

### 2.3 Gross Clinical Phenotype, Necropsy and Histopathological Investigation

Necropsy was performed on both affected kittens, and organ and tissue samples, including abdominal skin, were collected for histopathological investigation. Remaining tissues were freshly frozen and stored for further analysis. Selected regions of abdominal skin were fixed in 10% neutral buffered formalin for a minimum of 24 h prior to processing. Tissue processing included embedding in paraffin, sectioning via a microtome at 5 μm thickness, and staining with haematoxylin and eosin (H&E) before cover‐slipping for microscopic examination.

### 2.4 DNA Isolation and Whole‐Genome Sequencing

All genomic locations and positions reported in this study are in reference to the F.catus_Fca126_mat1.0 genome assembly (GCF_018350175.1). Genomic DNA from the two affected kittens was isolated from fresh‐frozen cardiac tissue using the Qiagen DNeasy Blood and Tissue kit, following the manufacturer's protocol. Whole‐genome sequencing (WGS) of both affected kittens was performed at Azenta Life Sciences (South Plainfield, NJ, USA) using Illumina NovaSeq 150 bp paired‐end reads, achieving an average of ×30.6 and ×30.3 coverage (for each kitten). Raw data processing, including quality control, read alignment, and variant calling was conducted utilizing the Whole Animal Genome Sequencing (WAGS) pipeline [31]. The functional effects of identified variants were annotated using Ensembl's variant effect predictor (VEP) [32] in conjunction with the NCBI *Felis catus* annotation release 105 for the F.catus_Fca126_mat1.0 genome reference assembly.

### 2.5 Variant Filtering

Genetic variants identified in the two IF‐affected kittens were compared against a control cohort of 100 publicly available cat genomes representing genetically diverse domestic cat breeds and mixes (Table S1). Variant filtering was performed using bcftools 1.17 [33] to identify variants unique to the two cases, under the assumption that the disease‐causing allele would be absent from the control cat cohort. Variants present in one or more control cats were excluded from further analysis. The remaining private variants were then prioritized based on their predicted impact on gene transcripts, with only those annotated as having a ‘high’ or ‘moderate’ effect retained for downstream analysis. To assess the potential disease relevance of these variants, gene names corresponding to the prioritized variants were evaluated using varelect [34], a phenotype‐based variation prioritization tool, which ranks genes with disease‐causing likelihood by giving them a score indicating the strength of association between that gene and specific phenotypic search terms, in this case ‘ichthyosis’. Genes with a varelect score ≥ 10 were considered potentially disease‐associated and selected for further investigation. Concurrently, the mapped sequence reads from both affected kittens were manually inspected using integrative genomics viewer (IGV) [35] to identify any variants occurring in genes previously associated with ARCI in other domestic animal species [1, 3, 4, 5, 6, 7, 8, 9, 10, 25, 26, 36, 37, 38] as reported by the Online Mendelian Inheritance in Animals (OMIA) [39] (Table S2).

### 2.6 Targeted Genotyping and Population Testing

Sanger sequencing was used to confirm the presence of the candidate variant *ABCA12*:XM_019838638.2:c6926delC in the two affected kittens and to screen for the deletion in a broader cohort of domestic cats. DNA from 140 unrelated cats representing 18 different breeds and random‐bred mixes was already available from prior, unrelated studies. These DNA samples were obtained from surplus or discarded blood collected during routine clinical procedures at the Purdue University Veterinary Hospital or from buccal swabs and blood samples collected under Purdue University IACUC protocol no. 1901001840. In all cases, sample collection was performed with signed owners' consent. PCR amplification of a 540 bp fragment (or 539 bp in the case of the deletion allele) spanning the variant site was performed using KOD Xtreme Hot Start DNA Polymerase (Millipore‐Sigma) and primers 5′‐ACTTGCAGACACAGAGTGAGG‐3′ (Primer F) and 5′‐TGCCCACATCACATTTGTTCAC‐3′ (Primer R) using standard conditions; primers were designed with primer3plus 3.3.0 [40]. Amplicons were treated with ExoSAP‐IT PCR Cleanup Reagent (Thermo Fisher Scientific) and submitted for Sanger sequencing (Eurofins Genomics, Louisville, KY, USA). Resulting sequences were analyzed with sequencher 5.4.6 (Gene Codes Corp.).

## 3 Results

### 3.1 Gross Clinical Phenotype

**FIGURE 1:**
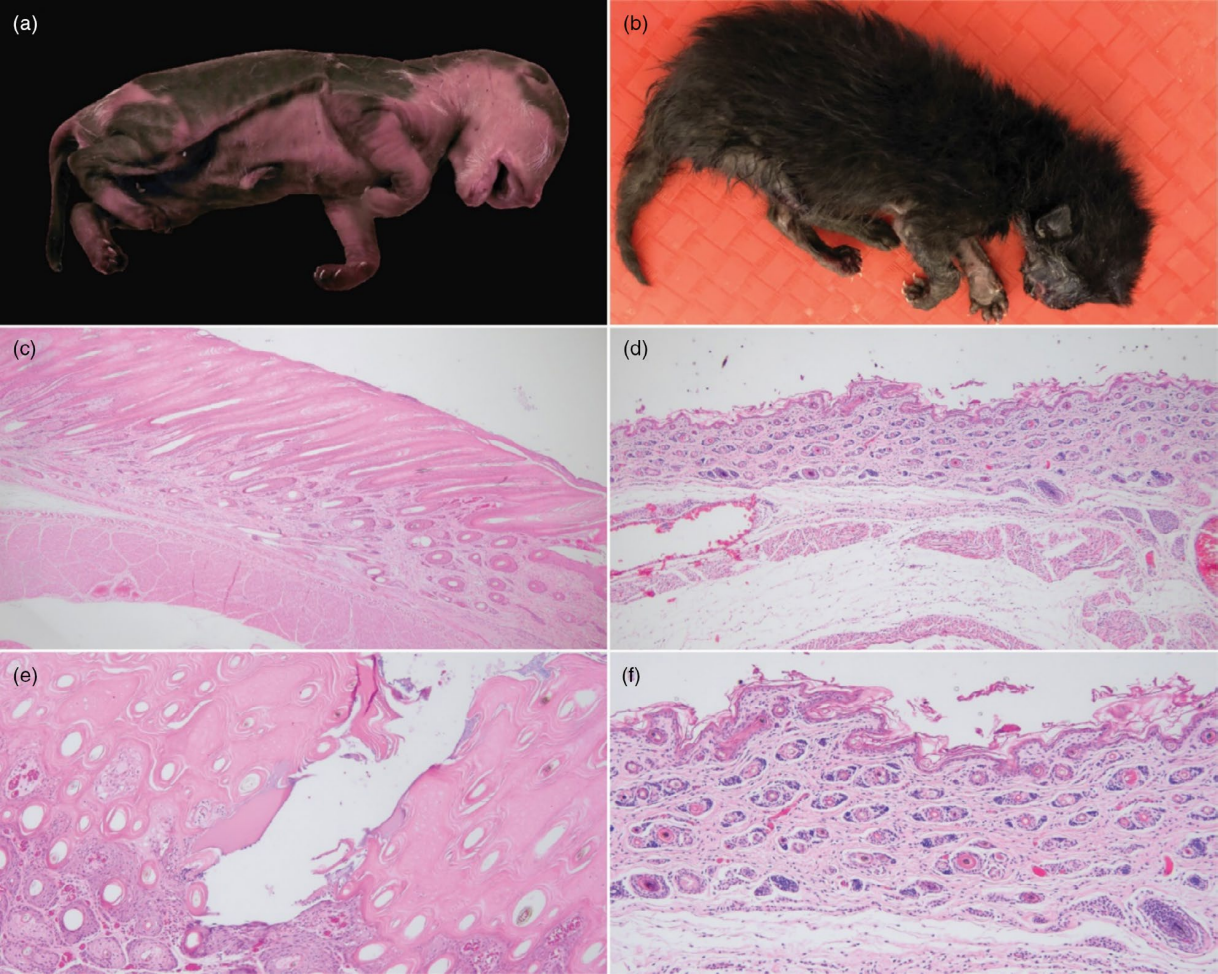
Ichthyosis fetalis in a kitten. (a) Gross photo of one of two affected kittens. Kitten is sparsely haired with a diffuse leathery appearance and multifocal areas of scaling. (b) Gross photo of an unaffected, unrelated 5‐day‐old control kitten, with typical haired integument. (c) Low magnification (×4) view of the abdominal skin of affected kitten, which is representative of process. Section highlights diffuse compact orthokeratotic hyperkeratosis with normal granular cell layer. (d) Low magnification (×4) view of the abdominal skin of neonatal kitten in (b) with a normal basket weave stratum corneum. (e) High magnification (×10) view of the abdominal skin of affected kitten, highlighting a keratin fissure associated with neutrophilic inflammation and embedded cocci within the dense keratin layers. (f) High magnification (×10) view of the abdominal skin of neonatal kitten in (b) with a normal basket weave stratum corneum. (c–f) Haematoxylin & eosin.

Both cases, deceased female newborn kittens, were moderately autolyzed (weights 60 and 68 g) with crown‐to‐rump measurements ranging from 12–12.5 cm. Based on these lengths, their gestational age was estimated at approximately 55 days (5–8 days premature) [41]. A viscous white fluid was present in the oral cavity of each neonate. The kittens were sparsely haired, with fine hairs observed on the limbs and dorsal neck, with facial vibrissae present. The skin surface appeared dry and leathery, with multiple small fissures exposing the underlying pink dermis. In one kitten, angular flakes of white keratin surrounded the fissures, predominantly within the abdominal skin. Both kittens also exhibited marked swelling of the oral cavity, including visibly red and swollen tongue, lips and gums (Figure 1a). The ears appeared underdeveloped, with small, folded pinnae and an immature external ear canal. It is noted that the sparse hair coat and underdevelopment/immaturity of the ears could be influenced either by the underlying disease or by the slightly premature gestational age. In contrast, the control specimen, a deceased, unrelated, unaffected 5‐day‐old kitten, demonstrated typical haired integument (Figure 1b).

### 3.2 Histopathological Findings

Histopathological investigation revealed that the lungs of both kittens were multifocally collapsed and atelectatic. Many of the alveoli contained variable amounts of macrophages, eosinophilic granular debris, bacterial bacilli and desquamated keratinocytes, findings consistent with perinatal aspiration pneumonia. The epidermis of both IF‐affected kittens was markedly thickened with 1–2 mm of compacted lamellar orthokeratotic hyperkeratosis with embedded hair shafts (Figure 1c). Deep fissuring of the SC, with fracturing and clefting of the dense orthokeratin, was rarely observed (Figure 1e). Aggregates of cocci were multifocally associated with superficial layers of keratin and rarely visualized within follicular infundibula. The dermis was densely cellular with developing compound folliculosebaceous units. Evidence of thermal injury was not present in all cutaneous sections examined. The diffuse epidermal hyperkeratosis was consistent with congenital ichthyosis, which in severe cases can be lethal. In particular, skin lesions were consistent with IF seen in neonatal calves [15]. For comparison, the dermis from the unaffected kitten is shown as a control (Figure 1d,f).

### 3.3 Genetic Analyses

**FIGURE 2:**
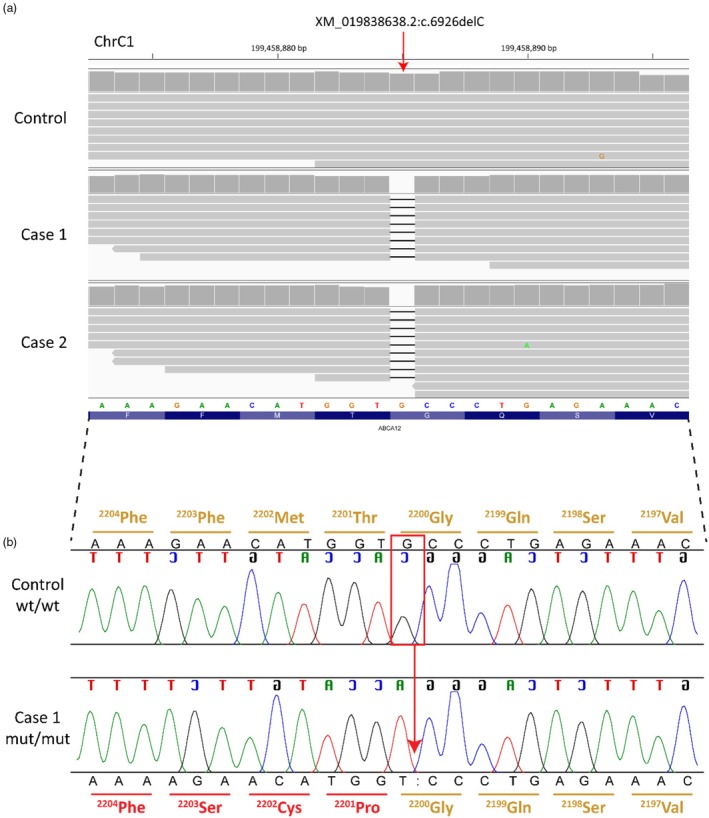
Identified *ABCA12* 1 bp frameshift deletion XM_019838638.2:c6926delC in cats with ichthyosis fetalis. (a) integrative genomics viewer (IGV) screenshot of the short‐read sequence alignments of a control unaffected cat and two affected cats (cases 1 and 2) demonstrates the 1 bp deletion (red arrow). *ABCA12* is on the reverse strand. (b) Sanger sequencing electropherograms for the reverse complement sequence showing the *ABCA12*:c6926delC (red box and arrow) in one wild‐type (WT) unaffected control cat and one IF‐affected kitten. The correctly translated amino acid sequence is shown in gold, and the frame‐shifted codons are marked in red. Sequence carried out to the stop codon can be seen in Figure S1. Case 2 appeared the same as Case 1. Complementary nucleotides representing the coding sequence are shown backwards (XP_019694197.1:p.Pro2201fsTer9).

The genomes of both affected kittens were sequenced, achieving average coverages of ×30.6 and ×30.3. Private variant analysis was conducted by comparing these genomes to the genomes from a control cohort of 100 cats, representing 16 breeds and 49 random‐bred individuals. Both dominant and recessive modes of inheritance were considered. Filtering for private, protein‐altering variants associated with ichthyosis in the two affected kittens identified a single potential pathogenic variant in *ABCA12*, a gene well‐established as causative for spontaneously occurring IF in humans and cattle [1, 17, 26] (Table 1 and Table S3). Both affected kittens were homozygous for a unique 1 bp deletion in exon 44 of the 53 annotated *ABCA12* exons (XM_019838638.2:c.6926delC; Figure 2a). This deletion is predicted to cause a frameshift in the coding sequence of *ABCA12* (ABCA12:p.Pro2201fsTer9), introducing a premature stop codon that truncates approximately 15% of the wild‐type (WT) open reading frame (Figure S1). Functional prioritization using varelect assigned *ABCA12* a score of 98.35, the highest score allotted for all 136 genes analyzed. This gene list included homozygous (*n* = 28 genes) and heterozygous (*n* = 108 genes) variants; note that genes could contain variants in multiple transcripts and were not analyzed if they were uncharacterized or lacked known homologs in other species.

**TABLE 1:** Results of variant filtering in two ichthyosis fetalis–affected kitten littermates against 100 control genomes. VarElect search term ‘ichthyosis’ score ≥ 10; VarElect score *ABCA12* = 98.35.

Filtering step	Homozygous variants	Heterozygous variants
Private variants shared between both cases	26,292	43,305
Protein‐changing private variants shared between both cases	84	539
Private protein‐changing variants shared between both cases located in functional candidate genes for similar phenotypes in other species (VarElect score ≥ 10)	1	0

The homozygous *ABCA12* frameshift variant identified through WGS was confirmed in both affected kittens by PCR and Sanger sequencing (Figure 2b). To assess the presence of this variant in the broader cat population, we genotyped an additional 140 cats representing various breeds and random‐bred mixes. All 140 individuals were homozygous for the WT allele, indicating that the variant is absent or extremely rare in the general cat population.

## 4 Discussion

In this study, we investigated two female neonatal kitten littermates initially presumed to be welfare/neglect cases, with the suspicion of thermal injuries. On gross examination, both kittens were sparsely haired and exhibited dry, leathery skin with multiple fissures and clefting of the compact orthokeratin, along with distinct craniofacial abnormalities, including eclabium, ectropion, flattened nose and rudimentary ears. While the characteristic extensive fissuring and diamond‐shaped keratin plaques commonly reported in IF appeared less pronounced in these feline cases, other hallmark features were present. These included sparsely haired skin surfaces, thickened and leathery skin, and underdeveloped pinnae and lips—features consistent with those described in bovine cases of IF [15, 42].

Histological examination of both kittens revealed diffuse, compact orthokeratotic epidermal hyperkeratosis, a characteristic feature of IF previously described in bovine cases. Notably, fewer of the fissures described within the bovine cases were seen both grossly and in histological section within these feline cases. This difference could be a result of sampling site, species‐associated variation, or simply the density of keratin at the time of death, as fissures are more likely to occur in the thickest regions of cornified epidermis or with increased friction and movement. Similar to IF in cattle, entrapped and embedded hair shafts were also documented within these kittens. Taken together, the combination of gross and histological findings was consistent with IF as described in other mammals [1, 25, 26].

Given the well‐established function of *ABCA12* in epidermal lipid transport and its strong association with the IF phenotype in other species, the identified frameshift variant is likely to be causal of the severe congenital ichthyosis observed in these kittens. To date, to the best of the authors' knowledge, only a single case of ichthyosis has been reported in a domestic cat—an Abyssinian kitten described in an abstract without subsequent peer‐reviewed publication [43]. That case presented with diffuse erythema and shiny abdominal skin, findings that are not consistent with the IF phenotype. Therefore, the present report represents not only the first documented case of IF associated with a pathogenic *ABCA12* variant in the domestic cat, but also the first genetic characterization of any form of ichthyosis in this species.

Pathogenic variants in *ABCA12* are associated with a spectrum of ARCI, including HI, CIE and LI, depending on the type and location of the variant. Homozygous or compound heterozygous truncating and predicted loss‐of‐function variants in *ABCA12* consistently result in the severe HI phenotype [19], while missense variants are more commonly associated with the milder phenotypes of CIE or LI (OMIM 601277) [18, 19, 20]. Like other members of the ABCA transporter family, the ABCA12 protein consists of two ATP‐binding cassettes and two transmembrane domains, each composed of six hydrophobic membrane‐spanning helices [44]. ABCA12 functions as a lipid transporter in epidermal keratinocytes that, when defective, leads to impaired skin lipid barrier formation [13].

The *ABCA12* frameshift variant identified in both affected kittens, ABCA12:p.Pro2201fsTer9, is predicted to result in a premature stop codon at the position normally encoding amino acid residue 2208 (Figure S1). Translation of the altered transcript is therefore expected to produce a truncated protein that terminates within the region normally forming the second transmembrane domain. As a result, the altered protein would lack approximately 15% of the C‐terminal sequence, including the second ATP‐binding cassette domain (UniProt M3WB04) [13, 20, 45]. Recessive truncating variants within these conserved regions of *ABCA12* lead to the severe harlequin ichthyosis phenotype in humans [19, 20], providing strong evidence for the pathogenicity of the ABCA12:p.Pro2201fsTer9 feline variant. These findings reinforce the functional importance of the transmembrane and ATP‐binding domains of ABCA12 and demonstrate that homozygous loss‐of‐function variants result in the IF phenotype in domestic cats.

One limitation of this study was the inability to assess gene expression to confirm the predicted loss‐of‐function effect of the *ABCA12* frameshift variant. Although fresh‐frozen tissue from both affected kittens was available, dermal tissue was not included, and the samples were not collected in a way to maintain mRNA or protein integrity. Future studies evaluating the functional consequence of the *ABCA12*c6926delC variant at the transcript or protein level are needed to fully understand its biological impact.

The absence of the identified *ABCA12* variant in a diverse cohort of unrelated domestic cats suggests that this is a rare allele which most likely appeared recently and is confined to this family. Given that the affected kittens were found as strays, it is plausible that the *ABCA12* frameshift deletion arose spontaneously and is identical by descent in both cases as a result of extreme inbreeding. Such consanguineous matings are relatively common in feral cat populations, increasing the likelihood of rare, homozygous recessive variants that can manifest deleterious or hereditary disorders [46].

## 5 Conclusions

We identified a recessive loss‐of‐function allele in the feline *ABCA12* gene in two neonatal kitten littermates diagnosed with IF, representing the first documented case of IF in the domestic cat. Consistent with IF phenotypes observed in other mammalian species, the gross clinical presentation included leathery skin, multiple fissures, craniofacial malformations, and marked epidermal hyperkeratosis. The absence of this pathogenic variant in a diverse cohort of unrelated domestic cats suggests that it is a rare allele, which is likely to have arisen recently and manifested homozygously in offspring due to extreme inbreeding. Increased awareness among veterinarians is warranted to consider this disease in the differential diagnosis of feline dermatopathies. However, given the rarity of this variant in the broader cat population, routine screening is not currently recommended. These findings support the importance of ABCA12 in mammalian species for lipid transport and expand the mutational spectrum associated with IF.

## Author Contributions

**Jeanna M. Blake:** conceptualization, data curation, formal analysis, investigation, methodology, software, visualization, writing – review and editing, writing – original draft. **Melissa P. Swan:** conceptualization, investigation, methodology, visualization, writing – review and editing. **Kari J. Ekenstedt:** conceptualization, formal analysis, investigation, methodology, project administration, supervision, writing – original draft, writing – review and editing.

## Funding

The authors have nothing to report.

## Conflicts of Interest

The authors declare no conflicts of interest.

## Supporting information


**Appendix S1:** vde70043‐sup‐0001‐AppendixS1.zip.

## Data Availability

All raw whole‐genome sequencing data are available from SRA under the accession no. PRJNA1290419 and sample accession nos SAMN49920859 and SAMN49920860. Accessions also are given in Table S2.
