# Supplementary material for: ABCA12 Frameshift Deletion in Domestic Cats With Ichthyosis Fetalis
Source: Vet Dermatol. 2025 Dec 15;37(3):365–74. doi: 10.1111/vde.70043 (PMC13167644; doi:10.1111/vde.70043)
Supplement: Supplementary file 1 — Appendix S1: vde70043‐sup‐0001‐AppendixS1.zip. [file VDE-37-365-s001.zip › vde70043-sup-0001-TablesS1-S2.docx]

**Supporting Information**

**TABLE S1.** Accession numbers of 102 cat genome sequences used in this study.

| **Sample ID** | **Breed** | **Study accession** | **Sample accession** | **Remark** |
| --- | --- | --- | --- | --- |
| 13711 | DSH (not pure-bred) | PRJNA1290419 | SAMN49920859 | Affected with IF |
| 13712 | DSH (not pure-bred) | PRJNA1290420 | SAMN49920860 | Affected with IF |
| K0748 | DSH (not pure-bred) | PRJEB7401 | SAMEA112465974 |  |
| K0749 | DSH (not pure-bred) | PRJEB7401 | SAMEA112465975 |  |
| K0763 | DSH (not pure-bred) | PRJEB7401 | SAMEA113550736 |  |
| K0764 | DSH (not pure-bred) | PRJEB7401 | SAMEA113550737 |  |
| K0766 | Bengal | PRJEB7401 | SAMEA113550738 |  |
| K0770 | Not pure-bred (European domestic long hair) | PRJEB7401 | SAMEA115420742 |  |
| K0772 | Ragdoll | PRJEB7401 | SAMEA115420740 |  |
| K0773 | Maine coon | PRJEB7401 | SAMEA115420745 |  |
| K0774 | DSH (not pure-bred) | PRJEB7401 | SAMEA115420743 |  |
| K0775 | DSH (not pure-bred) | PRJEB7401 | SAMEA115420744 |  |
| K0776 | DSH (not pure-bred) | PRJEB7401 | SAMEA115420741 |  |
| K0777 | DSH (not pure-bred) | PRJEB7401 | SAMEA115420739 |  |
| K0778 | DSH (not pure-bred) | PRJEB7401 | SAMEA115420737 |  |
| K0782 | Bengal | PRJEB7401 | SAMEA115420746 |  |
| K0783 | DSH (not pure-bred) | PRJEB7401 | SAMEA115420738 |  |
| K0784 | Domestic medium-length hair (not pure-bred) | PRJEB7401 | SAMEA115420747 |  |
| K0790 | British shorthair | PRJEB7401 | SAMEA115994775 |  |
| K0797 | European shorthair | PRJEB7401 | SAMEA115994775 |  |
| K0798 | Neva Masquerade | PRJEB7401 | SAMEA115994775 |  |
| K350 | Norwegian forest cat | PRJEB7401 | SAMEA5885912 |  |
| K443 | Maine coon | PRJEB7401 | SAMEA1061682 |  |
| K450 | DSH (not pure-bred) | PRJEB7401 | SAMEA2785958 |  |
| K452 | Bengal | PRJEB7401 | SAMEA1061681 |  |
| K468 | Egyptian Mau | PRJEB7401 | SAMEA14502946 |  |
| K469 | Egyptian Mau | PRJEB7401 | SAMEA1061683 |  |
| K471 | DSH (not pure-bred, from USA) | PRJEB7401 | SAMEA1061684 |  |
| K472 | DSH (not pure-bred, from USA) | PRJEB7401 | SAMEA1061685 |  |
| K473 | DSH (not pure-bred, from USA) | PRJEB7401 | SAMEA1061686 |  |
| K484 | Bengal | PRJEB7401 | SAMEA5885917 |  |
| K485 | Bengal | PRJEB7401 | SAMEA5885918 |  |
| K486 | Bengal | PRJEB7401 | SAMEA5885919 |  |
| K488 | Toyger | PRJEB7401 | SAMEA5885925 |  |
| K494 | Maine coon | PRJEB7401 | SAMEA5885913 |  |
| K499 | unknown | PRJEB7401 | SAMEA1061687 |  |
| K500 | Burmese | PRJEB7401 | SAMEA5885920 |  |
| K502 | British shorthair | PRJEB7401 | SAMEA1061688 |  |
| K509 | Bengal | PRJEB7401 | SAMEA5885921 |  |
| K510 | DSH (not pure-bred) | PRJEB7401 | SAMEA104694019 |  |
| K511 | DSH (not pure-bred) | PRJEB7401 | SAMEA1061799 |  |
| K513 | British shorthair | PRJEB7401 | SAMEA5885914 |  |
| K514 | British shorthair | PRJEB7401 | SAMEA5885915 |  |
| K515 | Persian | PRJEB7401 | SAMEA1061689 |  |
| K516 | Maine coon | PRJEB7401 | SAMEA5885928 |  |
| K517 | British shorthair | PRJEB7401 | SAMEA5885916 |  |
| K518 | DSH (not pure-bred) | PRJEB7401 | SAMEA5885922 |  |
| K519 | Maine coon | PRJEB7401 | SAMEA5885923 |  |
| K520 | Bengal | PRJEB7401 | SAMEA5885926 |  |
| K522 | Bengal | PRJEB7401 | SAMEA14502947 |  |
| K528 | Persian | PRJEB7401 | SAMEA5885983 |  |
| K529 | Persian | PRJEB7401 | SAMEA5885984 |  |
| K530 | Persian | PRJEB7401 | SAMEA5885985 |  |
| K531 | Persian | PRJEB7401 | SAMEA5885986 |  |
| K532 | DSH (not pure-bred) | PRJEB7401 | SAMEA5885924 |  |
| K533 | Not pure-bred (Bombay?) | PRJEB7401 | SAMEA5885927 |  |
| K544 | Domestic long hair (not pure-bred) | PRJEB7401 | SAMEA5885929 |  |
| K548 | DSH (not pure-bred) | PRJEB7401 | SAMEA5885930 |  |
| K549 | DSH (not pure-bred) | PRJEB7401 | SAMEA5905717 |  |
| K553 | DSH (not pure-bred) | PRJEB7401 | SAMEA7853381 |  |
| K556 | DSH (not pure-bred) | PRJEB7401 | SAMEA7853382 |  |
| K557 | Peterbald | PRJEB7401 | SAMEA13314551 |  |
| K560 | DSH (not pure-bred) | PRJEB7401 | SAMEA8952636 |  |
| K563 | DSH (not pure-bred) | PRJEB7401 | SAMEA6085996 |  |
| K564 | DSH (not pure-bred) | PRJEB7401 | SAMEA7853383 |  |
| K566 | Maine coon | PRJEB7401 | SAMEA7376281 |  |
| K570 | DSH (not pure-bred) | PRJEB7401 | SAMEA7376282 |  |
| K573 | Domestic long hair (not pure-bred) | PRJEB7401 | SAMEA7376283 |  |
| K578 | Domestic long hair (not pure-bred) | PRJEB7401 | SAMEA7376284 |  |
| K580 | DSH (not pure-bred) | PRJEB7401 | SAMEA7376285 |  |
| K581 | DSH (not pure-bred) | PRJEB7401 | SAMEA7376286 |  |
| K583 | DSH (not pure-bred) | PRJEB7401 | SAMEA7853384 |  |
| K584 | DSH (not pure-bred) | PRJEB7401 | SAMEA7853385 |  |
| K591 | Turkish van | PRJEB7401 | SAMEA8609184 |  |
| K596 | British shorthair | PRJEB7401 | SAMEA8609185 |  |
| K600 | Maine coon | PRJEB7401 | SAMEA8609188 |  |
| K601 | Maine coon | PRJEB7401 | SAMEA8952637 |  |
| K602 | DSH (not pure-bred) | PRJEB7401 | SAMEA8609186 |  |
| K610 | Siberian | PRJEB7401 | SAMEA8952638 |  |
| K620 | Ragdoll | PRJEB7401 | SAMEA8952639 |  |
| K626 | DSH (not pure-bred) | PRJEB7401 | SAMEA8952640 |  |
| K627 | DSH (not pure-bred) | PRJEB7401 | SAMEA8952641 |  |
| K629 | DSH (not pure-bred) | PRJEB7401 | SAMEA8952642 |  |
| K646 | Maine coon | PRJEB7401 | SAMEA8952643 |  |
| K649 | Maine coon | PRJEB7401 | SAMEA14502948 |  |
| K650 | Maine coon | PRJEB7401 | SAMEA13314552 |  |
| K654 | DSH (not pure-bred) | PRJEB7401 | SAMEA13314553 |  |
| K689 | Bengal | PRJEB7401 | SAMEA112203056 |  |
| K708 | Oriental shorthair | PRJEB7401 | SAMEA13314554 |  |
| K709 | Bengal | PRJEB7401 | SAMEA14502949 |  |
| K711 | DSH (not pure-bred) | PRJEB7401 | SAMEA14502950 |  |
| K714 | Maine coon | PRJEB7401 | SAMEA14502951 |  |
| K718 | Bengal | PRJEB7401 | SAMEA14502952 |  |
| K719 | British shorthair | PRJEB7401 | SAMEA14502953 |  |
| K724 | Abyssinian cat | PRJEB7401 | SAMEA112203057 |  |
| K727 | DSH (not pure-bred) | PRJEB7401 | SAMEA112203058 |  |
| K734 | DSH (not pure-bred) | PRJEB7401 | SAMEA112203059 |  |
| K745 | DSH (not pure-bred) | PRJEB7401 | SAMEA112203060 |  |
| K746 | DSH (not pure-bred) | PRJEB7401 | SAMEA112203061 |  |
| S19190 | Burmese | PRJNA288177 | SAMN05980350 |  |
| S19194 | Burmese | PRJNA343392 | SAMN05980373 |  |
| S19197 | Burmese | PRJNA343392 | SAMN05980372 |  |

Abbreviations: DSH, domestic short hair

**TABLE S2.** Results of OMIA search for genetic variants causing ARCI in other mammals.

| **OMIA ID** | **Phenotype** | **Species** | **Gene** |
| --- | --- | --- | --- |
| OMIA:002238-9913 | Ichthyosis, ABCA12-related | *Bos taurus* | *ABCA12* |
| OMIA:002368-9615 | Ichthyosis, ABHD5-related | *Canis lupus familiaris* | *ABHD5* |
| OMIA:003011-9615 | Ichthyosis, ALOXE3-related | *Canis lupus familiaris* | *ALOXE3* |
| OMIA:002243-9913 | Ichthyosis, DSP-related | *Bos taurus* | *DSP* |
| OMIA:002450-9913 | Ichthyosis, FA2H-related | *Bos taurus* | *FA2H* |
| OMIA:001415-9615 | Hyperkeratosis, epidermolytic | *Canis lupus familiaris* | *KRT10* |
| OMIA:001980-9615 | Ichthyosis, NIPAL4-related | *Canis lupus familiaris* | *NIPAL4* |
| OMIA:001588-9615 | Ichthyosis, PNPLA1-related | *Canis lupus familiaris* | *PNPLA1* |
| OMIA:002659-9615 | Ichthyosis, non-epidermolytic, SDR9C7-related | *Canis lupus familiaris* | *SDR9C7* |
| OMIA:001973-9615 | Ichthyosis, SLC27A4-related | *Canis lupus familiaris* | *SLC27A4* |
| OMIA:000546-9615 | Ichthyosis, TGM1-related | *Canis lupus familiaris* | *TGM1* |

**TABLE S3 (see separate Excel file).** Private homozygous and heterozygous variants in the two sequenced kittens affected by ichthyosis fetalis (sheet 1). Private variants were obtained by filtering out all variants that were present at least once in 100 cat control genomes. This resulted in 29,292 homozygous private variants (sheet 2) and 43,305 heterozygous private variants (sheet 3) shared between both cases. Variants predicted to have a "high" or "moderate" effect on resulting transcript were considered to be protein-altering. This resulted in 84 protein-altering homozygous private variants (sheet 4) and 539 protein-altering heterozygous private variants (sheet 5). (Note: Variants are listed multiple times if they have predicted effects on more than one transcript, and not every gene is annotated or has a known homologue). One private variant was shared between both cases and was located in a directly related functional candidate gene for similar phenotypes in other species (VarElect score ≥ 10; sheet 6).

Download

CLUSTAL O(1.2.4) multiple sequence alignment

WT MASQFHQLRVLVWKNWLGVKRQPLWTLVLILWPVIIFIILAITRTKFPPRAKPTCYLAPR 60

Mut MASQFHQLRVLVWKNWLGVKRQPLWTLVLILWPVIIFIILAITRTKFPPRAKPTCYLAPR 60

************************************************************

WT NLPSTGFFPFLQTLLCDTDSKCKDTPYGPQDLLRRKGINDALFKDSEILRKSSNLEKDSN 120

Mut NLPSTGFFPFLQTLLCDTDSKCKDTPYGPQDLLRRKGINDALFKDSEILRKSSNLEKDSN 120

************************************************************

WT LSLQSTKVPERRHTSPATVFPSTSSNLESMGTDTFNGSQILARILGLEKLLKQNSTSEDI 180

Mut LSLQSTKVPERRHTSPATVFPSTSSNLESMGTDTFNGSQILARILGLEKLLKQNSTSEDI 180

************************************************************

WT RRELCDSYPDYIAHYAFTWTTLGKNIFNRFCLSNMTFLESSLHELTNQFSQISSDPNNQK 240

Mut RRELCDSYPDYIAHYAFTWTTLGKNIFNRFCLSNMTFLESSLHELTNQFSQISSDPNNQK 240

************************************************************

WT TVFQEMVKVLSFFSQVQEQTAVWQLLSSFPSVFQNETTLSNLLDVLRNANSALLVVQKVY 300

Mut TVFQEMVKVLSFFSQVQEQTAVWQLLSSFPSVFQNETTLSNLLDVLRNANSALLVVQKVY 300

************************************************************

WT PRVTTNEGFKTLHKSVKHLLYTLDSPAQGGSGNTTHVWSEEDEQLLSPSSLAAQLLILEN 360

Mut PRVTTNEGFKTLHKSVKHLLYTLDSPAQGGSGNTTHVWSEEDEQLLSPSSLAAQLLILEN 360

************************************************************

WT FEDALLNISADSPYIPYLACVRNVTDNLARGSQENLRLLQSIISFKISFLQNGFYEDYLS 420

Mut FEDALLNISADSPYIPYLACVRNVTDNLARGSQENLRLLQSIISFKISFLQNGFYEDYLS 420

************************************************************

WT SVPEVVKSKLSQLRNLTELLCESETFSSIERMCQLSDMNFGNLCEESAFHVQLLEAAEIG 480

Mut SVPEVVKSKLSQLRNLTELLCESETFSSIERMCQLSDMNFGNLCEESAFHVQLLEAAEIG 480

************************************************************

WT TEIATNLLYRDNILSKKLRDLLTGDPSKINLNMDWFLEQALQMNYLENITRLMPTIEAMM 540

Mut TEIATNLLYRDNILSKKLRDLLTGDPSKINLNMDWFLEQALQMNYLENITRLMPTIEAMM 540

************************************************************

WT HVNNSADASEKRGQLIEMFKNVELLKEELRRTTGMSSRSVDSLLAIPIPDSRAEIISRVF 600

Mut HVNNSADASEKRGQLIEMFKNVELLKEELRRTTGMSSRSVDSLLAIPIPDSRAEIISRVF 600

************************************************************

WT WLHSCDANMTYSKMEDVMKEFCNLSVPERSRQSYLIGLTLLHYLDIYNFTYKVFFPREDQ 660

Mut WLHSCDANMTYSKMEDVMKEFCNLSVPERSRQSYLIGLTLLHYLDIYNFTYKVFFPREDQ 660

************************************************************

WT KPVEKMIELFMRLKEILSQMASGTRPLLDKMSSLKQMHLPRSVPLTQAMYRSNRMNTPQG 720

Mut KPVEKMIELFMRLKEILSQMASGTRPLLDKMSSLKQMHLPRSVPLTQAMYRSNRMNTPQG 720

************************************************************

WT SFSTISQALCSQGITTEYLTALLPSSQRRKGNHTKDFLTYKLSKEQIASKYGIPTHTTPF 780

Mut SFSTISQALCSQGITTEYLTALLPSSQRRKGNHTKDFLTYKLSKEQIASKYGIPTHTTPF 780

************************************************************

WT CFSLYKDIINMPAGPVIWAFLKPMLLGKILYAPYTPITKAIMEKSNVTLRQLAELREKSQ 840

Mut CFSLYKDIINMPAGPVIWAFLKPMLLGKILYAPYTPITKAIMEKSNVTLRQLAELREKSQ 840

************************************************************

WT EWMDNSPLFMNSFHLLNQTIPMLQNTLRNPFVQVFVKFSVGLDAVELLKQIDELDILRLK 900

Mut EWMDNSPLFMNSFHLLNQTIPMLQNTLRNPFVQVFVKFSVGLDAVELLKQIDELDILRLK 900

************************************************************

WT LENSIDLIDHLNTLSSLTVNISSCVLYDRIQAAKSIDEMERQAKRLYKSNELFGSVIFKL 960

Mut LENSIDLIDHLNTLSSLTVNISSCVLYDRIQAAKSIDEMERQAKRLYKSNELFGSVIFKL 960

************************************************************

WT PSNRSRHRGYDSENVSLPPVIKYTIRMSLKTAQTTRSIRTKIWAPGPHNSPSHNQIYGRA 1020

Mut PSNRSRHRGYDSENVSLPPVIKYTIRMSLKTAQTTRSIRTKIWAPGPHNSPSHNQIYGRA 1020

************************************************************

WT FVYLQDSIERAIIELQTGRNSQEIAVQVQAIPYPCYMKDNFLTSVSYSLPIVLMVAWVVF 1080

Mut FVYLQDSIERAIIELQTGRNSQEIAVQVQAIPYPCYMKDNFLTSVSYSLPIVLMVAWVVF 1080

************************************************************

WT IAAFVKKLVYEKDLRLHEYMKMMGVNSCSHFFAWLIESIGFLLVTIVILIIILKFGNILP 1140

Mut IAAFVKKLVYEKDLRLHEYMKMMGVNSCSHFFAWLIESIGFLLVTIVILIIILKFGNILP 1140

************************************************************

WT KTNGFILFLYFSDYSFSVIAMSYLISVFFNNTNIAALIGSLIYIIAFFPFIVLITVEDEL 1200

Mut KTNGFILFLYFSDYSFSVIAMSYLISVFFNNTNIAALIGSLIYIIAFFPFIVLITVEDEL 1200

************************************************************

WT SYIVKVFMSLLSPTAFSYASQYIARYEEQGIGLQWENMYSSPVQDDTTSFGWLCCLILAD 1260

Mut SYIVKVFMSLLSPTAFSYASQYIARYEEQGIGLQWENMYSSPVQDDTTSFGWLCCLILAD 1260

************************************************************

WT SFIYFLIAWYVRNVFPGTYGMAAPWYFPVLPSYWKERLGCTDMKHEKSNGLMFTNIMMQN 1320

Mut SFIYFLIAWYVRNVFPGTYGMAAPWYFPVLPSYWKERLGCTDMKHEKSNGLMFTNIMMQN 1320

************************************************************

WT TNPSASKTSPEYMFPSNIEPEPKDLTVGVALHGVTKIYGSKIAVDNLNLNFYEGHITSLL 1380

Mut TNPSASKTSPEYMFPSNIEPEPKDLTVGVALHGVTKIYGSKIAVDNLNLNFYEGHITSLL 1380

************************************************************

WT GPNGAGKTTTISMLTGLFGASAGTIFVYGKDTKTDLHTVRKSMGVCMQHDVLFSYLTTKE 1440

Mut GPNGAGKTTTISMLTGLFGASAGTIFVYGKDTKTDLHTVRKSMGVCMQHDVLFSYLTTKE 1440

************************************************************

WT HLLLYGSIKVPHWTKKQLHEEVKRTLKDTGLYSHRHKRVGTLSGGMKRKLSISIALIGGS 1500

Mut HLLLYGSIKVPHWTKKQLHEEVKRTLKDTGLYSHRHKRVGTLSGGMKRKLSISIALIGGS 1500

************************************************************

WT RVVILDEPSTGVDPCSRRSIWDVISKNKTARTIILSTHHLDEAEVLSDRIAFLEQGGLRC 1560

Mut RVVILDEPSTGVDPCSRRSIWDVISKNKTARTIILSTHHLDEAEVLSDRIAFLEQGGLRC 1560

************************************************************

WT CGSPFYLKEAFGDGYHLTLTKKKTPNLSANTTCDTMAVTTMIRSHLPEAYLKEDIGGELV 1620

Mut CGSPFYLKEAFGDGYHLTLTKKKTPNLSANTTCDTMAVTTMIRSHLPEAYLKEDIGGELV 1620

************************************************************

WT YVLPPFSTKVSGAYLSLLRALDNGMGDLNIGCYGISDTTVEEVFLNLTKESQKDRDMSLE 1680

Mut YVLPPFSTKVSGAYLSLLRALDNGMGDLNIGCYGISDTTVEEVFLNLTKESQKDRDMSLE 1680

************************************************************

WT HLTQKKIGNSSTNGISTPDDLSVSSSSFTDRDDKILTRGERLKGFDLLLKKIMAILIKRF 1740

Mut HLTQKKIGNSSTNGISTPDDLSVSSSSFTDRDDKILTRGERLKGFDLLLKKIMAILIKRF 1740

************************************************************

WT HHTRRNWKGLIAQVILPIVFVTTAMGLGTLRNSSNSYPEIQISPSLYGTSEQTAFYANSH 1800

Mut HHTRRNWKGLIAQVILPIVFVTTAMGLGTLRNSSNSYPEIQISPSLYGTSEQTAFYANSH 1800

************************************************************

WT PSTKALVSAMWSFPGIDNMCLNISDPRCLTRGSLGKWNTSGEPITNFGVCSCSENIQECP 1860

Mut PSTKALVSAMWSFPGIDNMCLNISDPRCLTRGSLGKWNTSGEPITNFGVCSCSENIQECP 1860

************************************************************

WT KFNYSPPHRRTYSSQIIYNLTGHRLENYLISTANEFMQKRYGGWSFGLPLTKDLRFDITA 1920

Mut KFNYSPPHRRTYSSQIIYNLTGHRLENYLISTANEFMQKRYGGWSFGLPLTKDLRFDITA 1920

************************************************************

WT VPANRTLAKVWYDPEGYHSLPAYLNSLNNFLLRVNMSKYDAARHGIIMYSHPYPGVQDQE 1980

Mut VPANRTLAKVWYDPEGYHSLPAYLNSLNNFLLRVNMSKYDAARHGIIMYSHPYPGVQDQE 1980

************************************************************

WT QATMSSLIDILVALSILMGYSVTTASFVTYVVREHQTKAKQLQHISGIGVTCYWATNFIY 2040

Mut QATMSSLIDILVALSILMGYSVTTASFVTYVVREHQTKAKQLQHISGIGVTCYWATNFIY 2040

************************************************************

WT DMVFYLVPVAFSIGVIAIFKLPAFYSENNLGAVSLLLLLFGYATFSWMYLLAGLFHETGM 2100

Mut DMVFYLVPVAFSIGVIAIFKLPAFYSENNLGAVSLLLLLFGYATFSWMYLLAGLFHETGM 2100

************************************************************

WT AFITYVCINLFFGINSIVSLSVVYFLSKEKPNDPTLELISETLKRIFLIFPQFCFGYGLI 2160

Mut AFITYVCINLFFGINSIVSLSVVYFLSKEKPNDPTLELISETLKRIFLIFPQFCFGYGLI 2160

************************************************************

WT ELSQQQSVLDFLKAYGVEYPSETFEMDKLGAMFVALVSQGTMFFLLRLLINECLIKKLRL 2220

Mut ELSQQQSVLDFLKAYGVEYPSETFEMDKLGAMFVALVSQGPCSFCCGS------------ 2208

**************************************** *

WT FFRKFSSSPVVETIDEDEDVRAERLRVENGASEFDLVQLHRLTKTYQLIHKKIIAVNNIS 2280

Mut ------------------------------------------------------------ 2208

WT IGIPAGECFGLLGVNGAGKTTIFKMLTGDIIPSSGNILIRNKTGSLGHVDSHSSLVGYCP 2340

Mut ------------------------------------------------------------ 2208

WT QEDALDDLVSVEEHLYFYARIHGIPEKDIKDTVHKLLRRLHLMPYKDRATSLCSYGTKRK 2400

Mut ------------------------------------------------------------ 2208

WT LSTALALIGKPSILLLDEPSSGMDPKSKRHLWRIISEEVQNKCSVILTSHSMEECEALCT 2460

Mut ------------------------------------------------------------ 2208

WT RLAIMVNGRFQCIGSLQHIKSRFGRGFTVKVHLKNTKVSMEALTRFMQLHFPKTYLKDQH 2520

Mut ------------------------------------------------------------ 2208

WT LSMLEYHVPVTAGGVANIFDLLETNKTALNITNFLVSQTTLEEVFINFAKDQKSYESADA 2580

Mut ------------------------------------------------------------ 2208

WT SSQGSTISVDSQDDRMES 2598

Mut ------------------ 2208

**FIGURE S1.** Protein alignment XP_019694197.1 (wild-type, WT) and mutant (Mut) sequence. The 1 bp frameshift deletion is predicted to translate eight erroneous amino acids (red) before encountering a stop codon; the latter is then predicted to truncate the resulting protein, with approximately 15% of the protein sequence lost.
